# The Horse’s Hoof1Abstract of Address delivered at the Montreal Veterinary College, February, 1890.

**Published:** 1890-06

**Authors:** Williamson Bryden


### THE HORSE'S HOOF.[1]

BY DR. WILLIAMSON BRYDEN.

THE variety of hoofs found under different circumstances and at different periods, and the information derived from a study of them in a state of nature and in domestication, reveal to us not only their susceptibility to favorable and unfavorable influences, but also the potency of different surroundings in accomplishing changes and modifications in their form, size and quality.

A proper appreciation of this is indispensable to the breeder and the veterinarian who would cultivate the hoof successfully, for in proportion to our comprehension of the subject will be our

[1] Abstract of Address delivered at the Montreal Veterinary College, February, 1890.

ability to overcome in a more natural way undesirable deviations in its growth, the result not only of congenital influences, but also of acquired defects as well. When this is supplemented by judicious surgical skill our ability to prevent, to improve, or even to overcome, defective forms and qualities will prove a most important accomplishment and lead to studies of the horse's locomotive organs that will be found of very great interest to students of comparative medicine and surgery, especially in branches such as orthopœdics and athletics.

As illustrating the effect of climate, soil and country in determining the character of the hoof, permit me to repeat what is told us by a celebrated African traveler (I think Sir Samuel Baker), that in Nubia and some districts on the banks of the river Nile, "asses have hoofs as large as our horses." This evidently comes from excess of moisture in the ground on which they have been brought up.

We are also aware that the hoofs of horses raised in mild climates and on low soft ground are large and round, with frogs covering one-third of the area of the sole, while those raised on dry and hilly countries have smaller hoofs from want of moisture and other causes.

The lesson this teaches when applied to an individual is, if a certain animal has hoofs that are small and dry, pasture him where the ground is soft and moist; if the hoofs are large and spongy, pasture on dryer upland. On some farms there may be ground suitable for certain kinds of grain, while other parts of the same farm may be quite unfit for such a crop. It is much the same in selecting suitable pasturage for our young horses.

If a breed of animals of excellent quality, pedigree and record, after intelligent tests by crossing, continue to produce a large percentage of progeny with poor hoofs and defective limbs in the North where the Winters are long, change them to farther South, where enough exercise all the year round can be assured, and the chances are that a desirable family of animals may be perpetuated instead of being allowed to gradually drift to decrepitude.

In every locality, even the most favorable, the restraints imposed by domestication yearly ruin many of our finest young animals, the want of *wear and tear* to the hoofs and exercise to the limbs being a most important factor in leading to such misfortunes.

Take, for example, a foal born a couple of months before the grass comes. It is confined in a pen, perhaps on dry plank floors, quite unsuitable for its tender little feet, which nature intended for a soft pasture. When Spring opens the weather may be dry, the soil poor or otherwise ill-suited to repair the mischief. Another period of five or six months' imprisonment is soon reached, and this is repeated till maturity.

When we reflect that we have not merely a single organism, but that each animal has four hoofs, and that these and the limbs above must reach maturity in a fairly symmetrical and unblemished condition, the necessity for intelligent watchfulness and supervision becomes apparent.

To explain this more fully : Take a yearling. He is well bred, looks well and is free from any noticeable lameness. On examining his feet, however, it is found that he places one hind foot evenly on the ground ; the other foot he places more on the toe. If this continues for a year or so during the growing period of his life, it is evident that among other changes in the hoofs, they will differ in shape, the heel of the one that bears most on the toe will be narrowest, and it is impossible that the limbs above can organize alike. Consequently their action or gait will be different, and also the predisposition to disease.

As an illustration of the effect on the limbs above when the weight is thrown irregularly on the hoof, or when pain or other disordered conditions of the foot reacts on them, I will describe a five years' old horse of good size and weight, as follows :

*Near Side.*—On the foreleg the hoof is of good size, but toes *in*, the outside heel is low, the fetlock hangs over it and has a small windgall outside and a small sprung ligament on the inner front. The ulna is *out* from the ribs, and the point of the shoulder is *in ;* the scapula is too far forward on the neck, which is full and bulges a little to the side.

*Off Side.*—On the off foreleg the hoof is of good size, but *toes out ;* the inside heel is low and the fetlock is over it. The ulna is close to the ribs and the scapula back on the shoulders.

Two such legs are unlike each other, and the gait is rocking and uneven.

The near foot describes a circle *out*, the off foot describes a circle *in*, and when trotting is liable to interfere with the other. Such a condition may be brought about when the foal suckles mostly on one side of the dam, especially in confined places, or it may be influenced by the way the attendant trims the hoofs of the young. If he is righthanded, the rasp is used from different points of vision and positions. The same applies to the horse-shoer. If he is righthanded, he levels the two feet for the shoe from opposite points, and also sets the shoe and drives it in the same way. On the near foot he drives the second nail on the outside first, and on the off foot the second nail on the inside first.

*Near Side Behind.*—The hoof is of fair size, but *toes in;* the outside heel is low, short and turned under; the fetlock is above this heel, and there is a puff above the sessamoid on the same side. Such a hoof binds at the coronet, and if scraped clean, where the hoof is white, it will show blood staining from the disturbance to the circulation by the tight coronet. The inside heel is long, well back and slopes under. When this has existed for some time, the fetlock will cockle, the angle of the tibia and femur will be lengthened; the crest of the ilium is *up* and *in;* the ischium is in and down, and the tail is drawn toward the left, unless it was previously twisted too far to the other side; dimples the result of atrophy, also appear over the hip articulation.

*Off Side Behind.*—The hoof is of fair size, but toes in; the outside heel is short and turned under; the fetlock is over the inside heel; the leg at the head of the metatarsal bone is bent inward, giving it a zigzag appearance when viewed from before or behind; the tendons behind the cannon bone do not overlap each other evenly; the summit of the calcis is pulled outward and causes a slight fullness below the base on the inner aspect; the stifle is out, and the crest of the ilium is not so prominent as the other, but the hip is wider and more fleshy and looks larger.

In the near hind foot just described we have one that predisposes or is liable to ordinary bone spavin, especially if the sole is concave and thick.

When, as I said before, the hoof is placed unevenly on the ground, either because there is a tender part or simply because it is most comfortable and has become a habit, the weight is unevenly distributed to the limb above, and it organizes in harmony with the defect in the hoof. If, for example, the hoof is placed on the ground in such a way that the metatarsal flexor has to do more than its share of work—when the animal is not too old—the gradual increased strain at its insertion leads to a projection at the seat of ordinary bone spavin. This may at first be healthy tissue, but when the inharmony in the organization of the limb has increased, from shortening of the muscle or other retrograde changes, the demand on the flexor or the parts of the hock involved is either too great or they are less able to meet this demand. True bone spavin, with its disastrous consequences to the animal, is the result.

One or both heels may shorten or grow thin between the bars and the external wall, or, if they are long, one or both may contract, sometimes uniformly; in other cases one more than the other. In contractions of this nature the bars are more upright and the commissures deep; the soft structures beneath are injured as with an ingrowing toe nail. This leads to atrophy, and even navicular disease in both fore and hind feet, or it may interfere with the circulation to the frog, and cause atrophy of it or even thrush.

In another form of contraction, familiarly known as hoof-bound, the foot has been held firmly in its grasp for a long time, and the interference with the circulation gives rise to another class of diseases, such as scratches. Owing to the disturbed condition of the extremities, the waste is imperfectly carried off by the bloodvessels and the thin skin behind the pastern performs this important function for them. In time chronic scratches supervene, and every time the animal is driven out they break out again—an excellent example of "disordered nutrition." This may continue still farther, especially in coarse-bred horses, and the whole foot become so disturbed that its secreting structures perform their function imperfectly, the walls become coarse and irregular, projections of horn are found transplanted and scattered over the heels and behind the pasterns; sometimes the disturbance is so great and long continued that the ergots and fetlock tufts of hair become mixed and grow in the form of a crust, reaching up and down the leg and familiarly known as rat-tails. Even the horny tissues called the chestnuts change as the foot becomes disordered.

When the hoof has acquired such a character that it affects adversely the structures it was intended to protect, the limb above is affected, at first indirectly and without any important structural change, and any change of gait it may have occasioned can be instantly corrected.

If the limb was held suspended for a sufficiently long time, a general atrophy would result; but if the animal is kept at work, and he puts the weight on his foot irregularly, changes take place in certain parts of the limb from too much work, while the other parts of the same limb may be affected adversely from too little, or from not being required to perform their proportion of work.

We are all familiar with what has been called strain of the flexor tendons. Here the commissures deepen until the animal cannot comfortably rest his weight on his heel; going on his toe causes it to be brought directly under the leg, to relieve them. This is accomplished principally through the flexors, which are overtasked and strained; the surrounding structures, from the foot to the carpus, and even higher, become hypertrophied, and in the brachial region there is atrophy, while the most conspicuous feature of all is their having become greatly shortened. It is evident that such a case can be successfully treated only when the hoof is recognized as the cause, atrophy of structures within the hoof being easiest overcome when the mechanical pressure it exerts is relieved.

This shortening of structures in connection with atrophies and hypertrophies reminds me of a blunder I made in an article to the *Veterinarian* in 1876.

In trying to explain the importance of the hoof as the chief source of derangements of the limbs above, I stated that "the tail is drawn to the side on which the muscles are strongest." This I have since discovered to be a mistake, the animal I was taking my observations from having a kind of congenital twist or bend in her tail to the off side too great to be overcome by the shortening on the atrophied side.

In the study of animal organization this is a most important matter, as without a knowledge of this fact our explanations of many cases of imperfect organization are simply mysterious phenomena, impossible of being explained scientifically. It is a most important factor, too, in bringing about that inharmonious muscular organization that gives us erratic gaits, equivocal locomotive action and many of those diseased conditions of the limbs to which horses are particularly liable. For example: When so-called tarsal tenotomy has afforded relief, the *tension* has not always been due to increase of the subjacent bone, but to retrograde changes and shortening of the structures divided.

We are all familiar with muscular relaxation the result of an injury, where the tail, for example, would be drawn *to the side on which the muscles are strongest;* but where we have atrophies, etc., the result of what may be called peripheral disturbances, we have in many cases shortening of structures instead of relaxation.

In examining for bone spavin one of the most important points is the heel. Place the horse's hind feet together on a level floor and measure the heel of each shoe. If they are alike in size and width at the heel, the foot of the spavined limb being smallest, more of the shoe will be exposed than on the sound foot. Of course this applies to other troubles of the leg as well, and is very important, as such a leg is always weaker than its fellow.

There are at least three kinds of hoofs peculiar to as many different forms of springhalt, and springhalt acquired during the growing period of the animal's life is generally more difficult to benefit than when acquired in adult life.

Anchylosis, specimens of bone spavin, etc., without any history, are mere curiosities, and every specimen to be of value ought to have been written up during life, and the hoofs, bones and ligaments preserved.

